# Enhancing Surgical Curriculum: Trainees' Perspectives on Laparoscopic Simulation and Assessment

**DOI:** 10.7759/cureus.77054

**Published:** 2025-01-07

**Authors:** Yousif Aawsaj, Jitendra Singh, Muhammad S Shamim

**Affiliations:** 1 Colorectal Surgery, Northumbria Healthcare NHS Foundation Trust, Newcastle upon Tyne, GBR; 2 General Surgery, Northumbria Healthcare NHS Foundation Trust, Newcastle upon Tyne, GBR; 3 Medical Education, Aga Khan University, Karachi, PAK

**Keywords:** assessment, certification, general surgery curriculum, laparoscopic training, simulation

## Abstract

Introduction

Laparoscopic simulation has been used in many curricula. The United Kingdom (UK) surgical curriculum lacks summative assessment for laparoscopic skills. This study explores surgical trainees’ perceptions of using simulated laparoscopic assessment as a summative tool in the UK.

Methodology

This was a semi-structured interview study conducted in person for 10 higher surgical trainees recruited voluntarily in the northern region of England. A thematic analysis was used to analyse the data.

Results

The findings generally showed positive perceptions among the trainees for simulated laparoscopic assessment. The trainees highlighted that the current assessments are formative and often subjective. Trainees suggested introducing summative assessment might fit with current changes in the national curriculum. The interviews also showed that simulated laparoscopic assessment would positively affect the trainees, curriculum, and patients. In addition, trainees expressed that the introduction of such a change should be staged and tailored to the training grade. However, the majority did not support making it part of *CCT *requirements. The trainees emphasised that implementing such a change can face challenges such as cost, tension between training and service provision, and culture change. The practicality of introducing simulated laparoscopic assessment was discussed in terms of skills to be assessed, fidelity, and progression signposting.

Conclusion

The study highlighted the trainees' perceptions about simulated laparoscopic assessment, which was generally positive, and raised issues regarding challenges in its implementation. Further discussion in surgical societies is required and in-depth research is needed before implementating simulated laparoscopic assessment for trainee certification.

## Introduction

Surgical training in the UK is undergoing a transition from a time-based to a competency-based framework, providing trainees more flexibility to achieve their surgical competencies [1]. However, the reduction in hands-on operative opportunities remains an issue in developing competent surgeons. A survey has shown that only one-fifth of the general surgical training programmes in the UK are adherent to the Joint Committee on Surgical Training (JCST) quality indicator in terms of operative exposure [2]. The ramifications of this scarce exposure will continue in these trainees' consultant lives and will put them in a disadvantageous position compared to their peers.

One of the ways to alleviate this problem is by increasing the role of simulation in surgical training and assessment. Based on the evidence on the use of simulation in surgical training, the American College of Surgeons (ACS) and Food and Drug Administration (FDA) have supported the role of simulation for surgical procedures during residency programmes in the USA [3,4]. The American Board of Surgery has made it mandatory for surgical trainees to complete a simulated laparoscopic course, Fundamentals of Laparoscopic Surgery (FLS), as part of the required competencies [5]. However, the Intercollegiate Surgical Curriculum Programme (ISCP) seems to be deficient in simulated training and assessment and most of the Procedure-based Assessments (PBAs) during surgical training in the UK are still generic; only a few indicate the type of surgical exposure - laparoscopic or open. In addition, there are limited speciality-specific PBAs, especially for lower and upper gastrointestinal surgery trainees [6]. With the current transformation of the UK surgical curriculum, simulation can be promoted to expand the hands-on exposure and training opportunities for the trainees.

Therefore, the aim of this study was to explore the perception of the surgical trainees on the current methods of assessment and on enhancing the use of laparoscopic simulation in training and assessment, both formative and summative. The study also explored the study participants’ thoughts about their experience of acquiring laparoscopic skills through simulation. The results from this study may help the decision-makers and trainers in designing an improved surgical training programme, improving the competencies of surgeons.

## Materials and methods

Research approach

A qualitative study design using semi-structured interviews was selected for this study, which involved the use of pre-determined open-ended questions (see the Appendices for the interview questionnaire) while also allowing for the flow of conversation like an open discussion [7]. In-person interviews were conducted with the study participants, as they are considered the gold standard for providing in-depth discussion and monitoring of responses and cues [8]. A pilot interview with a general surgery (ST6) trainee provided insights into the time frame required for conducting the interviews and allowed for the refinement of questions and their order for a smoother flow of conversation. Before the interviews, the interviewees were provided with a summary sheet of the study via email and a paper sheet during the interview. The interviewer and researcher roles were carried out by the first author to minimise confounding and in addition, participants and interviewees had similar exposure to the training pathway to further reduce reflexivity bias. As per Thematic Analysis sample size requirements, 15 interviews were agreed upon as the sample size. However, 15 trainees were invited but out of this 10 participants were chosen on the basis of their response and availability.

Ethical considerations

Ethical approval for the study was obtained from the School Student Project Ethics Committee (S-SPEC) at Keele University. The deaneries were informed via email about the upcoming interviews and a general overview of the project, but without disclosing the identity of the interviewees and the selection process. Informed consent was obtained from the interviewees to share their views and quotes from their conversations, and they were assured that the data would be kept confidential. 

Semi-structured interview design

A semi-structured interview included North East and Merseyside deanery general surgical trainees and trainers, with a sample of 10 trainees selected to represent the in-depth views of the surgical trainee population regarding the use of simulated laparoscopy for assessment. The schedule for each interview was agreed upon with the interviewees based on their availability, with an average time of 20-30 minutes allocated for each interview. 

Analysis methods

Recorded interviews were the sole source of data and no other method of data collection was used. The data was coded, interpreted, and presented to enrich the project and represent the interviewees’ views. The process of data analysis involved extracting the data from each recorded interview. A constant comparative technique was used, where the recording and transcript were constantly compared to ensure that all relevant information was extracted and compared to provide meaningful data that could be tabulated and compared. Deductive thematic analysis was chosen as the analysis method as it is a straightforward approach that best fits the type of data collected in this study.

## Results

A total of 10 face-to-face interviews were conducted with male and female trainees from various backgrounds and at different levels of training and experience (Table 1).

**Table 1:** Summary of demographic data of the interviewees. General Surgery speciality training starts at ST3 (Speciality Trainee Year 3) and trainees progress yearly for the next 5 years as ST4, ST5, and so on. Speciality Trainee Year 8 (ST8) marks the end of the training programme. GI: gastrointestinal

Variables	Category	Number of trainees
Age (years)	25-30	0
	31-35	6
	36-40	6
Gender	Male	6
	Female	4
Grade	ST3	1
	ST4	1
	ST5	3
	ST6	1
	ST7	2
	ST8	2
Year of graduation	2008	1
	2012	3
	2013	2
	2010	2
	2003	1
	2009	1
Speciality	Upper GI	5
	Lower GI	5
Country of graduation	UK	5
	Non-UK	5
Number of laparoscopic procedures	Less than 500	3
	500 to 1000	4
	More than 1000	3

Three themes with nine sub-themes were generated through content analysis of interviews (Table 2). 

**Table 2:** Summary of themes, sub-themes, and illustrative quotations. CCT: Certification of Completion of Training; PBAs: Procedure-based Assessments

Themes	Sub-themes	Quotations
Perception of current laparoscopic assessment in the surgical curriculum and introduction of a summative assessment tool	The current laparoscopic assessment methods in training	‘You are assessed based on PBAs and they are ok and easy to do. I think engagement from trainers tends to be quite low and tends to be more like a self-reflective tool. It is very rare to have a sit-down feedback session with the trainer unless they are very interested in training which is quite few and far between’’ (Interview 9).
	Perception of laparoscopic simulation in general	‘I think in more advanced techniques such as suturing and stapling simulation it could be very useful, but I am not sure if threading a mint polo has added much to me, and I cannot translate it in theatre’ (Interview 9).
	Laparoscopic simulation as an assessment tool	‘It is a useful tool to introduce. Compared to endoscopy, where we have a summative assessment, why not in laparoscopy?’ (Interview 1).
	Awareness of any available laparoscopic assessments	‘I am aware of LapPass (the Laparoscopic Passport) offered by the professional organisation ALSGBI (Association of Laparoscopic Surgeons of Great Britain and Ireland), but I do not think it is formal yet or an essential requirement; it is attended by trainees voluntarily’ (Interview 4).
Implications and barriers of introducing laparoscopic assessment	Implications of introducing laparoscopic assessment on stakeholders	‘Learning curve is very long without simulation and I think suturing is a classic example. Similarly, complex skills one may lose if they are not performed regularly. As a trainee, you will feel more confident in demonstrating these skills in certain operations, especially if you have been objectively assessed and certified. It would have a confidence-boosting effect’ (Interview 5).
	Barriers facing the introduction of laparoscopic assessment	‘Surgeons are hard to convince to change. So, pushing for change in a training program would not be an easy job' (Interview 3). ‘Cost, resources, and tight timings of the training program. I am not sure that the less-than-full-time trainees would be able to fit this in their list of tasks and competencies that they need to achieve’ (Interview 6).
Practicality of introducing laparoscopic simulation for assessment	Type of skills to be assessed in laparoscopic simulation	‘Basic skills should be assessed ideally in the early stages. In the later stages, assessment should be tailored towards specific procedures and specialty.’ (Interview 3). ‘Communication and interpersonal skills are a very important part of operating and should be taken as one package. I think these skills should be integrated into technical skills and we should assess trainees for them even though we are focusing on the operating side’ (Interview 4).
	Where does laparoscopic assessment fit in the curriculum?	‘It would be useful from the start of training, at the entry of ST3. For example, at ST3/4 you are expected to be competent at performing laparoscopic appendectomies and basic laparoscopic skills. Procedures you get as ST6 include lap colorectal resections and upper GI standards procedure’ (Interview 7). ‘I think by CCT you would be beyond the point of someone looking at your basic skills, but it might be useful in key stages of training such as ST4 to tick that box’ (Interview 5).
	Level of fidelity use in the laparoscopic assessment	‘I think it needs to be step-wise. For junior trainees, start with a box trainer and increase fidelity as you go through training for example, by the introduction of cadavers and focusing on core procedures such as lap cholecystectomy, lap appendectomy, and lap hernia repair. The issue would be the variability of simulators and variable quality, but the closest thing I can think of is cadaveric and video simulation as good as they can be; robotic virtual reality can offer a wide range of simulation but there is no haptic feedback so there will be a missing element of reality. I am not sure if any of the current simulators would replicate complex specialised procedures’ (Interview 5).

Theme 1: Perception of current laparoscopic assessment in the surgical curriculum and introduction of a summative assessment tool

The results of the study show a mixed perception of the current assessment in the surgical curriculum and the introduction of a summative assessment tool. The first part of the interview started with asking candidates about current assessment tools for operative skills generally and laparoscopic skills specifically. The general response from the trainees was that the current assessment tools, which are PBAs, are very subjective, trainers-dependent, and formative.

 'Current assessments are very subjective as they depend on the trainer and their relation with you or if they like you, so I feel it is biased as they might give you assessment that you do not deserve or vice versa. The problem with the current assessment is, blunt four levels. The highest level 4b implies that you dealt with complication but it does not specify what complication.' (Interview 2)

Participants were divided on the usefulness of the current assessment tools (PBAs), which were described as subjective and dependent on the trainer. Some participants believed that the current assessment tool was appropriate for the purpose, but most acknowledged the need for a more objective and standardised tool for assessment.

 'Most of the trainees become competent at the end of their training, but there is a need for an objective tool to standardise the competency. The current practice has objective tools such as PBA, but it is not formally standardised as a summative assessment.’ (Interview 1)

There were mixed views of the responses among the trainees; some agreed it is very useful to practice in simulated settings and it boosts confidence while operating on live patients. Others thought that although laparoscopic simulation provides a good framework to develop skills and enhance operative performance, it would never replace the practice and real patients. 

‘I think in more advanced techniques, such as suturing and stapling, simulation could be very useful, but I am not sure if threading a mint polo has added much to me, and I cannot translate it in theatre.’ (Interview 9)

‘It is always different when doing it in courses than doing it on a patient, and the difficulty is trying to recreate a real patient situation,** **but you will learn few skills** **such as getting familiar with the instruments. Doing it in a course does not mean that you can do it on patients, but it provides an excellent framework and ground that you can build up in your practice.’ (Interview 7)

In terms of laparoscopic simulation as an assessment tool, responses were generally positive. Many participants supported the introduction of summative assessment as a valuable tool for evaluating trainee progression, especially if similar tools had been successfully implemented in other areas like endoscopy and core courses.

‘There is room for having laparoscopic skills as a requirement for entry or progress, for example ATLS or CRISP needed to be passed to progress through training; so there is room to introduce such a laparoscopic course and pass it as an entry or progress requirement, especially in GI surgery.’ (Interview 5)

However, there were some challenges highlighted, such as the differences in trainee background and experience, the increasing focus on robotic surgery simulation, and the difficulty of standardising assessments. 

‘It is hard because trainees have different backgrounds and different training experiences - you might have trainees who have many years of trust-grade experience versus trainees who do not have this experience.' (Interview 3)

‘Like, look at robotic surgery - now there are hours and simulation you need to do as it is relatively new skills, and I think laparoscopic surgery, which is almost everywhere now, you are expected to perform lap surgeries as a part of standard care.’ (Interview 5)

The new curriculum was seen as a positive opportunity to introduce the change, with participants commenting that a structured program with a validated assessment tool could help in the acceleration of skill acquisition. 

‘I think it is useful,​ especially if it would be introduced in stages, such as early, intermediate, and advanced stage. Where you could add this form of assessment at the end of each stage and it would be a part of competencies that needed to be achieved before moving to the next stage.' (Interview 6)

‘The trainers’ confidence and trust will be more. I personally would be more confident in allowing a trainee to do a particular step in an operation** **who has had simulated training than one who has not tried it before.’ (Interview 4).

The level of entrustment between the trainer and the trainee was also expected to increase with the introduction of a summative assessment tool.

‘This is the whole point of introducing multiple consultant reports which fit with the new curriculum. I expect that similar to this proposal, something in the future will be introduced. For an example, assessors will come and assess you during an operation.’ (Interview 7)

Theme 2: Implications and barriers of introducing laparoscopic assessment

Despite the positive aspects, several barriers were identified that might prevent the introduction of laparoscopic assessment, such as cost, limited resources, limited time, and reduced investment in training. 

Participants also mentioned that training was often compromised by service pressure within the NHS, reducing the time available for training.

‘In the NHS, service always competed with training. Training through years is diminishing due to service pressure, rota cover, staffing. Training it is not seen as a part of the job anymore; it is seen as a privilege.’ (Interview 10)

Participants opined that the introduction of new assessments to the curriculum would face resistance from trainers as well as trainees, thereby highlighting the need for effective planning and resource allocation to ensure the successful implementation of a summative laparoscopic assessment tool.

‘Trainers' and trainees' resistant to change. Extra time and the cost with resources, all contribute to the barriers because time needs to be allocated for the trainees to practice and that would impact the service hugely because of the staff shortage.’ (Interview 8)

Theme 3: Practicality of introducing laparoscopic simulation for assessment

The majority of the participants in the interviews expressed support for incorporating laparoscopic simulation as an assessment tool at various stages throughout surgical training. They noted that assessments at different stages could demonstrate progress and identify trainees who may require additional laparoscopic training.

'At different stages of training, for an example, one can be at CT level and then to ST6, it can show the progress and highlight the trainees who are falling behind. For an example, in ST6 assessment if it shows that actually they are not where they are expected to be, then they can get extended training to get more laparoscopic experience or go to a job that is heavy in laparoscopic training.’ (Interview 3)

However, there was opposition to making laparoscopic simulation a requirement for obtaining a CCT. Respondents argued that at the CCT stage, it was expected that trainees were competent in their laparoscopic skills and that the emphasis should be on their decision-making abilities, prioritisation, and other non-technical skills. Also, they noted that while laparoscopic simulation may be useful at earlier stages of training, it may not be necessary to assess basic skills at the CCT level.

‘I think by CCT stage you would be beyond the point of someone looking at your basic skills, but it might be useful in key stages of training, such as ST4, ST6, to tick that box.’ (Interview 5) 

## Discussion

The study aimed to explore the views of surgical trainees regarding the role of current assessments in the UK surgical curriculum, particularly in relation to laparoscopic procedures. Rich data was gathered from the trainees about their views on the current assessment of laparoscopic procedures. The trainees also looked at the wider picture of the constant strain between training and service provision in the NHS.

The study participants represented a wide spectrum of surgical trainees, predominantly belonging to gastrointestinal (GI) surgery, across the program from level ST1 to ST8. 

The study found a degree of frustration among trainees with the current assessment approach, particularly with regards to PBAs, which are the only accredited in-training assessment tool at present. Although PBAs have the potential to provide objective and structured feedback, they are often seen as merely ticking a box and the variability of their educational effect is a concern. However, there is a lack of understanding regarding their summative or formative nature of assessment, and a concern for conflict of interest as, in many instances, the trainer can take the role of the assessor [9]. Surgery trainers and deaneries might need to clarify the role of PBAs in surgical curricula and consider them a summative tool for the assessment of laparoscopic skills.

Simulated laparoscopic assessments as part of different curricula are well established in many surgical training programs [5] and are seen as a positive alternative by trainees in this study. The current UK surgical curriculum is lagging in comparison to other programs in terms of the introduction of laparoscopic simulation as a training and assessment tool [6]. Interviewees showed a positive attitude toward introducing simulated laparoscopic assessments and compared laparoscopic skills to other similar skills such as endoscopy, where simulations have become a regular part of formative and summative assessments [10]. 

However, there are laparoscopic courses that are conducted throughout the year which are tailored to teach different levels of skills with the help of simulation and involve skill assessment. Awareness of any UK-based laparoscopic assessment was explored among the interviewees; none of the trainees have done it but most of the trainees have heard about LapPass® (The Laparoscopic Passport). LapPass® is the first laparoscopic assessment developed by an official organisation - the ALSGBI (Association of Laparoscopic Surgeons of Great Britain and Ireland, United Kingdom; London, UK)- to certify surgical trainees and recognise good performers. A survey was conducted for both trainers and trainees about the validity and usability of LapPass® has shown there is an element of face and content validity; although more research is needed to explore its predictive capability in the clinical setting, LapPass® potentially can be utilised as an assessment tool for the UK trainees pending further evaluation [11]. 

The literature supports the use of simulation for selection, certification, and credentials [12], although senior trainees in the study tend to be more conservative in their views. This was similar to the finding from another study [13]. Several studies have shown that general surgical trainees and trainers support the use of real patients rather than laparoscopic simulation for selection and progression assessments [14-17]. 

On the contrary, trainees in this study have expressed a positive attitude towards simulated laparoscopic assessment. This could be due to technological advancement as many of the studies not supporting simulation are a decade old, i.e., when low-fidelity-level simulations were used. Also, several relatively recent surveys have shown that trainer surgeons support using simulation for various assessments [18, 19]. This could be due to a shift in the thought process (comfort level) of the new generation of surgical trainees and the changing views of society towards digitalisation and use of technology, thereby warranting an in-depth investigation of the role of laparoscopic assessment in the surgical curriculum.

Despite the limitations of its limited scope, this study highlights a desire for laparoscopic simulation assessment among surgical trainees. A wider view from surgical trainees, which includes the perspectives of both higher and junior trainees, is required to gain a comprehensive understanding of the topic. Additionally, it is recommended that further discussions be held among deaneries and royal colleges to fully explore the topic. 

This study focused solely on trainees and did not include trainers, so it would be beneficial to conduct in-depth interviews with UK surgical trainers to gain their insight into the possibility of incorporating laparoscopic simulated assessment into the curriculum. This is especially relevant considering recent changes in the curriculum, making now an opportune time to examine this issue.

## Conclusions

There is broad recognition that simulated assessments provide a structured and objective assessment approach. However, there are concerns about cultural resistance to change, particularly among the current generation of surgical trainers. The study highlights the need for the surgical curriculum to address the role of laparoscopic assessment and to consider the potential benefits of incorporating simulated laparoscopic assessments. Given the current transformation of the curriculum, this is an opportune moment to have a more in-depth discussion on this topic. The findings suggest that the introduction of simulated laparoscopic assessment should be phased in and that it should be objective, structured, and fair. The ISCP may benefit from incorporating more simulated training and assessment, which is lacking at present.

## Appendices

Interview Questionnaire

Consent completed: Demographic: Age: Stage of training: Speciality: Gender: Place of graduation: Years since graduation: Number of laparoscopic procedures:

- Do you have any experience in laparoscopic simulation? Can you tell me what do you feel about that experience and implications on your following training? - What is your thought about the current laparoscopic assessment way for trainees? - Tell me about your thoughts on the current assessment of laparoscopic skills? How can it be improved if any improvements needed?

Laparoscopic simulation implications on the trainees

- What implications do you think it will have on the trainees’ confidence and future performance? - What if simulation can be a part of assessment at the end of the training? Say, a part of CCST (Certification of Completion of Speciality Training) assessment for example? - Could this be beneficial or not? Why? - What do you think the implications of using laparoscopic assessments on patient, trainees, trainers and the organisations? - Why do you think it is not yet established in the ISCP (Intercollegiate Surgical Curriculum Programme) (UK based curriculum)? - What is your thought about the best way of assessments in future? Cadaver? High fidelity? Low fidelity? Virtual reality?

Laparoscopic simulation as a tool of assessment

- What type of Laparoscopic skills do you think should be assessed? - What is your thought about using it to assess NOTES (Natural Orifice Transluminal Endoscopic Surgery)? - How do you think Laparoscopic skills can be assessed? - How do you think dealing with complications and unexpected events should be assessed?

Laparoscopic simulations as a tool for trainees’ progression

- At what stage do you think Laparoscopic assessment should be introduced? - How often do you think it should be performed? - Who the assessors should be? Any special characteristics? - What is your thought about simulated laparoscopic skills to be integrated in the curriculum? - Anything else that you may want to add in this discussion?
